# Individual-, home- and preschool-level correlates of preschool children’s sedentary time

**DOI:** 10.1186/s12887-020-1948-y

**Published:** 2020-02-07

**Authors:** Suvi Määttä, Hanna Konttinen, Rejane Augusta de Oliveira Figueiredo, Ari Haukkala, Nina Sajaniemi, Maijaliisa Erkkola, Eva Roos

**Affiliations:** 1 Folkhälsan Research Centre, Samfundet Folkhälsan, Topeliuksenkatu 20, 00250 Helsinki, Finland; 2 Faculty of Social Sciences, University of Helsinki, P.O.box 66, FI-00014 University of Helsinki, Helsinki, Finland; 3 Department of Food and Environmental Sciences, University of Helsinki, P.O. Box 66, FI-00014 University of Helsinki, Helsinki, Finland; 4 Faculty of Educational Sciences, University of Helsinki, P.O.box 66, FI-00014 University of Helsinki, Helsinki, Finland; 5 Clinicum, Department of Public Health, University of Helsinki, P.O.box 66, FI-00014 Helsinki, Finland

**Keywords:** children, preschool, home, parents, sedentary time, socioecological model

## Abstract

**Background:**

Prechoolers’ significant portions of sedentary time (ST) is a public-health concern due to its high prevalence and negative health consequences. However, few studies have explored correlates of preschoolers’ ST covering individual-, home- and preschool- factors within one study. The aim of this study was to identify the associations between multiple individual-, home- and preschool-level factors and preschoolers’ ST. In addition, it was studied how much individual-, home- and preschool-level factors explained the variance in children’s ST.

**Methods:**

A total of 864 children aged three to six, their parents and 66 preschools participated in the cross-sectional DAGIS study, which occurred between 2015 and 2016. The children wore an accelerometer for 1 week. Guardians, principals and early educators completed questionnaires covering the potential correlates of children’s ST, for example, temperament, practices, self-efficacy and regulations. Multilevel regression analyses were conducted in multiple steps; calculation of marginal and conditional R^2^ values occurred in the final phase.

**Results:**

Of the 29 studied correlates, the following factors remained significant in the final models. Being a boy (*p* < 0.001) and having high levels of surgency temperament (*p* < 0.001) were associated with lower ST. Regarding the home setting, frequent co-visits in physical activity (PA) places (*p* = 0.014) were associated with lower ST. Higher parental perceived barriers related to children’s outside PA (*p* = 0.032) was associated with higher ST. None of the preschool setting factors remained significant in the final model. Approximately 11% of the variance in children’s ST was attributed to factors related to the individual level whereas 5% was attributed to home-level factors; and 2% to preschool-level factors.

**Conclusions:**

This study identified a set of correlates of preschool children’s ST. Interventions aimed at reducing children’s ST should develop strategies targeting established correlates of preschoolers’ ST covering individual-, home- and preschool-level factors. The preschool-level factors included in this study explained little the variance in children’s ST. However, the included measures may not have captured the essential preschool-level factors that specifically influence children’s ST. Therefore, more studies are needed regarding potential preschool-level factors.

## Background

Preschool-age children, generally aged between three and five, spend about half of their waking hours in sedentary [1] . Sedentary time (ST) is defined as the time spent in sedentary behaviors (SB); SB is defined as any waking behavior characterized by an energy expenditure of less than or equal to 1.5 metabolic equivalents (METs) while in a sitting, reclining or lying posture [2]. ST can therefore consist of multiple different types of SBs (e.g. screen time, playing still and reading) that can accumulate throughout the week and across multiple settings. Higher ST in adulthood is associated with negative health consequences such as chronic disease outcomes and all-cause mortality, although high levels of daily physical activity (PA) may eliminate these risks [3–5]. The evidence of health consequences of overall ST in preschool-age is less conclusive [6]. However, as many other health behavior habits, SB habits are rooted in early childhood and tend to track later in life [7]. As preschool children spend significant proportions of their time in sedentary, it is relevant to study the factors associated with children’s ST [1, 8, 9]. For instance, studies have identified the role of home and preschool settings in influencing preschool children’s ST, stating that children in center-based care such as preschool are highly sedentary compared to children cared for at home; however, the opposite results have also been found [1, 10]. The difference in these results may relate to different ST measurement methods or cultural context of studies. This dissimilarity also underlines the importance of understanding which factors may cause variations in preschool children’s ST.

According to the socioecological models of health behaviors [11], multiple factors at a variety of ecological levels can influence ST. These correlates may be positioned at individual levels: for example, biological and temperament characteristics; or at environmental settings:such as home and preschool [11–13]. The environmental setting can consist of the physical environment (the type of available physical environment), social environment (the type of attitudes and beliefs of significant others) and, regarding preschool, also the organizational environment (the type of rules and regulations) [14]. Due to the early developmental stage of preschool children, children are highly receptive and dependent on their caregivers in their daily movement behaviors (e.g. ST, and PA) [15]. Caregivers in both of these settings, such as guardians, principals and early educators, can help to shape children’s possibilities to conduct movement behaviors through a variety of social environmental mechanisms, including encouragement, beliefs and attitudes, role modelling, rules and regulations, involvement and facilitation [16, 17]. Thus, it is important to understand which social environmental factors in both settings are associated with children’s ST.

Although the number of studies regarding correlates of preschool children’s ST has risen following Hinkleys’ et al review in 2010 [18], insufficient evidence prevents drawing conclusions about the correlates of ST. For instance, numerous studies have recognized that girls are more sedentary than boys. However, the influence of other child characteristics such as temperament on preschool children’s ST is less studied [1, 19]. Similarly, a recent review highlights the lack of knowledge about preschool social- and organizational level factors associated with children’s ST [20]. Slightly more knowledge exists of associations between social environment in the home setting and preschool children’s screen time as an indicator of ST [21]; however, the correlates of screen time and overall ST seem to be distinct [22]. Another relevant aspect of social environment is socioeconomic status (SES) and its associations with children’s behaviors [23]. Studies indicate associations between lower parental SES or lower neighborhood SES, and a higher risk of an unhealthier lifestyle and cardiovascular disease [24–27]. However, studies regarding the associations between parental or neighborhood SES and children’s ST are scarce, with contradictory findings, which can result from the variety of possible ways to measure SES [24, 28].

Many of these previous studies have also explored the influence of either preschool OR home; the influence of the combination of these settings is lacking. Following the principles of socioecological models, these settings interact with each other influencing children’s behavior [14]. Thus, a recent systematic review encourages the exploration of potential correlates across multiple levels of influence of socioecological model within one study [29]. Based on these above-mentioned considerations, this study aims to investigate which individual-, home- and preschool-level factors are associated with preschool children’s ST**.** In addition, this study aims to discover the extent to which individual-, home- and preschool-level factors explain variance in children’s ST.

## Methods

### Study design and population

The cross-sectional DAGIS (Increased Health and Wellbeing in Preschools) survey was conducted in eight municipalities in Southern and Western Finland in 2015 and 2016. More detailed information about the study purposes and the flow chart of participation in this study can be read elsewhere [30, 31]. Briefly, the main recruitment criterion for the randomly invited preschools was the existence of at least one preschool group with children aged between three and six. In the chosen municipalities, 86 preschools (51% of those invited) agreed to participate in the study, and organized the recruitment of families and children. A total of 983 guardians (27% of contacted guardians) gave written permission for their child to participate in the study. Implementation of study procedures required participation of at least 30% of children in at least one preschool group in a preschool; 91 guardians had a child in a preschool with less than the 30% participation rate and were thus excluded from the study. In addition, 28 children did not have any data. A total of 864 (24% of invited) children from 66 preschools (39% of invited preschools) participated in the study. The University of Helsinki Ethical Review Board in the Humanities and Social and Behavioral Sciences approved the study procedures (6/2015, approved on 25th February, 2015).

### Measures

#### Children’s sedentary time

Children wore an Actigraph W-GT3X accelerometers (Actigraph, LLC, Fort Walton Beach, Florida) for one week. Actigraphs have been validated and used extensively as an objective measure of PA and ST for different age groups and in different contexts [32–34]. Research group set the accelerometers on the children’s right hip during the first day of measurement in the preschool. After data collection, the epoch length was set at 15 s. Periods of 10 min or more at zero accelerometer counts were considered non-wear times and were excluded [35]; however, possible nap-times were not excluded. The analyses applied the Evenson ST cut-point (0–25 counts per 15 s) [36], a renonwned estimate of free-living ST [37, 38]. We set the following accelerometer wear-time criteria for children in the analyses: at least 600 min of wear time each day; at least four days with at least one weekend day during a measurement week. Because wearing hours varied between children, the variable was adjusted for the wearing hours so that the overall ST minutes was divided by the the total accelerometer wearing time and multiplied by 60 to create outcome variable expressed as average minutes per hour (min/h). Consequently, the measure used in this study indicates the average children’s ST minutes in one hour.

#### Individual level

Research group measured the height, weight and waist circumference of participating children in the preschool. These measurements allowed calculation of children’s body mass index (BMI) by dividing the weight in kilograms by the square of the height in meters. Each guardian reported their child’s age and gender in the guardian’s questionnaire. Guardians were given a separate questionnaire regarding children’s temperament, which asked guardians to consider their children’s reactions to 36 situations in the past six months. The questionnaire is based on a short form of the Child Behavior Questionnaire for children aged three to seven [39], which has demonstrated satisfactory internal consistency, criterion validity and longitudinal stability and provides a comprehensive assessment of children’s temperament [39]. The separate 36 items are typically coded into three summary scores to illustrate separate temperament domains: negative affectivity, effortful control and surgency. A child with a high level of negative affectivity is typically angry and fearful with a lowered mood and low soothability. A child with a high level of effortful control enjoys low-intensity activities and is characterized by increased attentional capacities, inhibitory control. A child with high surgency levels is impulsive, enjoys situations with high stimulus intensity and feels comfortable in social situations [39].

#### Home level

One guardian in each family completed the guardian’s questionnaire that covers attitudes, beliefs, self-efficacy, practices and availability of items related to children’s energy balance-related behaviors (EBRBs). This questionnaire also had multiple questions about family SES. Guardians could complete the questionnaire either online or on paper. The questions were based on formative work of the research group, the previously validated questions and items specific to the Finnish context [40–43]. The questionnaire included items related either to screen time, ST or PA because, as yet, only a limited number of associations between ST and potential correlates among preschool children have been recognized. In addition, we chose a mothers’ education as the indicator of parental SES because it seems to be the strongest, reliable and most consistent determinant of children’s health behaviors and childhood obesity [24, 44, 45]. Table 1 presents all the items used in this study. The selection of these home setting measures for this study was based on the following criterias: a) the included measures capture social environment related to children’s ST extensively, b) the measures are measured similarly (or almost similarly) in preschool level, and c) the measures are relevant to measure based on the previous research findings.

**Table 1:** Summary of measures included in the analysis

Level of influence	Variable	Survey items in the questionnaire	Scale, coding, and Cronbach alpha if relevant	Descriptive	N
Individual level	Gender		1 = girl, 2 = boy		
	Age		Continuous	Mean 4.74 SD 0.89	864
	BMI			Mean 15.87 SD 1.42	809
	Surgency	Based on established codings (see Putnam & Rothbart, 2006). E.g., likes going down high slides or other adventurous activities; often rushes into new situations;	Scale from extremely untrue of your child (1) to extremely true of your child (7), Cronbach alpha 0.80	Mean 4.69 SD 0.09	751
	Negative affectivity	Based on established codings (see Putnam & Rothbart, 2006). E.g., gets angry when s/he cannot find something s/he wants to play with; becomes upset when loved relatives or friends are getting ready to leave following a visit	Scale from extremely untrue of your child (1) to extremely true of your child (7), Cronbach alpha 0.76	Mean 3.70 SD 0.88	751
	Effortful control	Based on established codings (see Putnam & Rothbart, 2006). E.g., when drawing or coloring in a book, shows strong concentration; prepares for trips and outings by planning things s/he will need.	Scale from extremely untrue of your child (1) to extremely true of your child (7), Cronbach alpha 0.74	Mean 5.20 SD 0.72	751
Home level					
Parental beliefs and attitudes	Parental self-efficacy for activating child for physical activity (PA) ^1^	How confident are you that you could do the following? a) I can get my child to do something physically active no matter how busy his/her day is. b) I can get my child to be physically active no matter what the weather is like. c) I can get my child to be physically active even if he/she wants to use electronic devices. d) I can get my child to be physically active even if he/she wants to stay inside. e) I can get my child to be physically active even when there are no other children playing outside.	The answer options ranged from strongly disagree (1) to strongly agree (5). The statements were combined and divided by the number of items. Cronbach alpha 0.62	Mean 4.05 SD 0.75	789
	Parental self-efficacy for limiting children’s screen time ^1^	How much do you agree or disagree with the following statements? a) I am concerned about my child’s use of electronic devices. b) I find it difficult to limit my child’s screen time if he/she does not want and starts whinging. c) I find it difficult to restrain myself from using electronic devices when my child is around.	The answer options ranged from strongly disagree (1) to strongly agree (5). The statements were combined and divided by the number of items. Cronbach alpha 0.46	Mean 1.9 SD 0.74	793
	Parental attitude for importance of PA	How much do you agree or disagree with the following statement? a) It is important for me to make sure my child gets enough PA each day.	The answer option ranged from strongly disagree (1) to strongly agree (5). In the analyses, the ‘somewhat disagree’ was treated as a reference category as there were no ‘strongly disagree’ answers.	Mean 4.34 SD 0.70	797
	Parental attitude toward societal pressures for screen time ^1^	How much do you agree or disagree with the following statements? a) Sport as a hobby and the related costs (e.g., equipment, materials, subscription fees) are too expensive. b) There is pressure from society to purchase and use different electronic devices. c) It is important for my child to learn how to use electronic devices, because I am not very good at using them myself.	The answer options ranged from strongly disagree (1) to strongly agree (5). The statements were combined and divided by the number of items. Cronbach alpha 0.34	Mean 2.8 SD 0.80	791
	Parental perceived barriers related to children’s outside PA^1^	How much do you agree or disagree with the following statements? a) Poor weather limits my child’s opportunities to play outside. b) I find it difficult to let my child be physically active outside as I always have to be there to supervise him/her.	The answer options ranged from strongly disagree (1) to strongly agree (5). The statements were combined and divided by the number of items. Cronbach alpha 0.36	Mean 2.23 SD 0.92	792
	Parental beliefs of unhealthy energy balance-related behaviors (EBRBs) as a problem	To what extent do you think the following matters are generally a problem among 3-to-6-year-old children? a) Being overweight. b) Excessive screen time. c) Physical inactivity	The answer options ranged from not at all a problem (1) to a very big problem (5). The statements were combined and divided by the number of items. Cronbach alpha 0.75	Mean 3.27 SD 0.65	800
Parenting practices	Rules for limiting children’s screen time	Do you have limits on how much time your child can spend: a) Watching television. b) Using other screens.	The three answer options were “yes,” “no,” and “don’t have the equipment.” This question was recoded so that “don’t have the equipment” answers [for the television *N* = 20 (2.5%) and for other equipment *N* = 15 (2%)] were set as missing values.	Yes(0) = 74%, *n* = 565 No (1) = 26%, *n* = 197	762
	Parental practice for allowing child run around inside	How much do you agree or disagree with the following statement? a) My child is allowed to run around and be physically active inside our house.	The answer options ranged from strongly disagree (1) to strongly agree (5).	Mean 4.38 SD 0.90	796
	Parental screen use in front of children	Approximately how many hours a day do YOU usually use electronic devices during leisure time when your child is around a) During weekdays. b) During weekends.	Answer options (per day): 1 = none, 2 = less than 30 min, 3 = 30 min–1 h, 4 = 1–2 h, 5 = 3–4 h, 6 = 5 h or more. The items were recoded so that 1 = less than 30 min, 2 = 30–60 min, 3 = more than 60 min, and they were combined into one variable. This sum variable was recoded into three categories: 1 = less than 30 min, 2 = 30–60 min, 3 = more than 60 min.	Less than 30 min (1) = 33%, *n* = 263 30–60 min (2) = 40%, *n* = 320 More than 60 min (3) =27%, *n* = 213	796
	Parental PA in front of children	During the past week, how often did your child see you being physically active?	Answer options: never (1), 1–2 times (2), 3–4 times (3), 5–6 times (4) and daily (5).	Mean 2.6 SD 1.19	798
	Frequent co-visits in PA places	How often does your child go to the following places with at least one adult in the family? a) Nature/forest. b) Park and playground. c) Own yard. d) An indoor facility	The original answer options (less than once a month (1), 1–3 times per a month (2), 1–2 times a week (3), 3–4 times a week (4), 5–6 times a week (5) and daily (6)) were recoded to average weekly visits together at least with one parent in parks, forests/nature, own yard and indoor sport facilities.	Mean 7.8 SD 3.8	799
	Mother’s educational level	The highest educational attainment on a seven-item list: (1) comprehensive school (2) vocational school (3) high school (4) bachelor’s degree or college (5) master’s degree and (6) licentiate/doctor (7) other.	The response options were re-organized into three groups: a low education was defined as comprehensive schooling (usually from ages 7–16) to secondary education (usually ages 16–19); a medium level refers to a bachelor’s degree; and a high education as at least a master’s degree.	Low education 30%, *n* = 251 Medium education 41%, *n* = 353 High education 29%, *n* = 250	853
Preschool level					
Principals’ beliefs and attitudes	Principals’ personal interest in health	How much do you agree or disagree with the following statement? a) I am personally interested in nutrition, PA, and health.	The answer options ranged from strongly disagree (1) to strongly agree (5). In the analyses, the ‘either agree or disagree’ was treated as a reference category as there were no strongly disagree or somewhat disagree answers.	Mean 4.63 SD 0.55	58
	Principals’ attitude about the importance of children’s PA	How much do you agree or disagree with the following statement? a) In my opinion, it is important to increase children’s PA in preschool.	The answer options ranged from strongly disagree (1) to strongly agree (5).	Mean 4.77 SD 0.53	58
	Principals’ attitude about the importance of decreasing children’s sedentary time	How much do you agree or disagree with the following statement? a) In my opinion, it is important to decrease children’s sedentary time in preschool.	The answer options ranged from strongly disagree (1) to strongly agree (5).	Mean 4.63 SD 0.66	58
	Principals’ self-efficacy for influencing children’s behaviors	To what extent can you, as the principal, impact the following? a) How physically active the children are. b) The number of electronic devices in the preschool. c) The use of electronic devices in the preschool.	The answer options ranged from not at all (1) to very much (5). Cronbach alpha 0.65	Mean 3.92 SD 0.75	58
	Principals’ beliefs of unhealthy EBRBs as a problem	To what extent do you think that following matters are generally a problem among 3-to-6-year-old children? a) Being overweight; b) Excessive screen time; c) Physical inactivity	The answer options ranged from not at all a problem (1) to a very big problem (5). The statements were combined and divided by the number of items. Cronbach alpha 0.70	Mean 3.04 SD 0.62	58
Organizational policies and practices	Frequency of visits in PA places	How often does your preschool group visit the following places: a. Forest/place for a nature trip. b. Park. c. Neighborhood sports facilities or Gym. d. Other indoor facility for PA. Partly open-ended question so that early educator openly reported the times, but selected the frequency from options: weekly, monthly, yearly.	Recoded so that average weekly level visits in nature/forests, parks, gym (not own) or neighborhood sport facilities was calculated	Mean 1.74 SD 1.35	142
	Screen time policy	Do you have instructions on the following themes in your preschool: a) Permitted screen time for the children. b) Supervision of the children’s use of electronic devices. c) Staff’s use of own electronic devices. d) In-service training for the staff on screen time. e) Bringing electronic devices to the preschool on a toy day (e.g., a tablet)	The original answer options (1 = no instructions, 2 = oral instructions, 3 = own written instructions and 4 = other written instructions) were summed up so that a maximum score 20 means that all asked items are other written instructions; the possible range in scores was between 4 and 20.	Mean 8.55 SD 2.50, measured minimum 5 and maximum 15	58
	Guidance for families policy	Do you have instructions on following themes in your preschool: a) Guidance for families on children’s PA (indoors and outdoors). b) Guidance for families on screen time.	The original answer options (1 = no instructions, 2 = oral instructions, 3 = own written instructions and 4 = other written instructions) were summed up so that a maximum score 8 means that all asked items are other written instructions; the possible range in scores was between 2 and 8.	Mean 3.27 SD 1.66, Measured minimum 2 and maximum 8	58
	Healthy PA policy	Do you have instructions on the following themes in your preschool: a) Children’s daily amount PA indoors and outdoors. b) Limiting children’s sedentary behavior. c) Staff’s practices in encouraging PA. d) Planned physical education for children. e) In-service training for staff on children’s PA (indoors and outdoors). f) Ensuring sufficient outdoor play time regardless of the weather conditions. g) Limiting children’s PA/running outdoors. h) Limiting children’s PA/running indoors.	The original answer options (1 = no instructions, 2 = oral instructions, 3 = own written instructions and 4 = other written instructions) were summed up so that a maximum score 32 means that all asked items are other written instructions; the possible range in scores was between 4 and 32.	Mean 19.55 SD 5.37 Measured minimum 10 and maximum 32	58
	Active play possibility during free play	Do children always have the possibility to play actively during free play time? a) In the group facilities. b) Elsewhere than in the group facilities	Combined measure so that Yes = possible to play actively at least in one place, or no = no possible at all. Due to answer distribution, this measure was recoded so that 1 = no at all possible, 0 = others	14,5% had no active play possibility, *n* = 21 groups	142
	Preschool neighborhood socioeconomic status (SES)	The score for the SES of each preschool was calculated using database information on a) income (median population income in the area logarithmically transformed), b) educational level (percentage of over 18-year-olds whose highest educational level was a master’s degree or beyond), and c) area unemployment rate. The unemployment rate was coded inversely to get higher values for lower unemployment rates.	The preschool SES score for each preschool neighborhood was calculated by taking the mean value of the z scores on income, educational level and unemployment rate. The score was then divided into tertiles representing low, middle, and high preschool SES.	31,5% in low SES, *n* = 21 30,7% in middle SES, *n* = 21 37,8% in high SES, *n* = 24	66

^1^ Based on loadings in the factor analysis

*PA* Physical activity, *EBRBs* Energy balance-related behaviors, *SES* Socioeconomic status, *SD* Standard deviation

#### Preschool level

Multiple questionnaires were used to measure the preschool setting. Principals were asked to complete a web-based questionnaire covering both the rules and regulations related to EBRBs in their preschool and their own attitudes and beliefs about children’s EBRBs. Because each preschool group in a preschool can define their own practices, we had an additional questionnaire for each preschool group that more precisely measured their group-based practices related to children’s EBRBs. One early educator in each preschool group completed this paper-based questionnaire. The questions in both questionnaires were based on formative work of the research group, the previously validated questions and items developed suitable for the Finnish context [46–48]. The preschool neighborhood SES was obtained from Statistics Finland. This grid database contains coordinate-based statistical data calculated on a map grid within a one-kilometer radius from the participating preschools. Table 1 presents all the items used in this study. The selection of these home setting measures for this study was based on the following criterias: a) the included measures capture social or organisational environment related to children’s ST extensively, b) the measures are measured similarly (or almost similarly) in home level, and c) the measures are relevant to measure based on the previous research findings.

### Covariates

Covariates in the analyses were ‘Children’s average attendance at preschool’ and ‘Study season’. ‘Children’s attendance at preschool’ was a composite score of the following questions in the guardians’ questionnaires: ‘How many days per week does your child attend preschool? ’ and ‘How many hours per day does your child usually attend preschool? ’. The average children’s daily attendance hours in preschool (hours/day) was formed by combining these measures. The ‘Study season’ measure was divided into three categories: 1 = September–October, 2 = November–December, and 3 = January–April.

### Statistical analyses

The descriptive statistics, factor analysis of sum variables and multicollinearity tests were conducted using the SPSS statistical program version 24 (SPSS Inc., Chicago, IL, USA). Multicollinearity was tested using tolerance and variance inflation factors. The results indicated no issues with multicollinearity.

Linear regression models examined the associations between explanatory factors and children’s ST, with analyses conducted in multiple steps. First, the main effects of all the potential explanatory factors was individually examined. These regression models were conducted in Mplus version 7.14. (Muthen & Muthen, 2017). MLR (maximum likelihood with robust standard errors) was used as an estimator in the analyses. These models accounted for clustering of children within families with participating siblings, preschools and preschool groups. If the responses were nested in the same higher-level unit, such as children attending the same preschool, multilevel design were applied. In these multilevel models, children were set as the first level unit, and the preschools or preschool groups as the second level unit. In addition, each individual level variable was group-mean centered [49, 50].

In the second phase, all the explanatory factors indicating association with ST (*P*-value < 0.10) from the previous phase were included in the same model. For this model, we calculated the marginal and the conditional R^2^ as per Nakawaga and Schilzeth (2013). Marginal R^2^ is the variance explained by fixed factors in the model, and conditional R^2^ is the variance explained by fixed and random factors [51]. After estimating the model, the “sum square” of each fixed factor (independent variables) was used to calculate the explained variance for each independent variable. First, the percentage of explained variance between all fixed factors was calculated. As the fixed factors explained only 29.8% of the variance, variance explained was estimated to the full model.

## Results

Of the 864 participating children, 773 children had valid accelerometer data for the analyses. Of them, 48% were girls. The average age of children with accelerometer data was 4 years and 7 months (SD = 0.86).On average, the children had 28.1 min of ST per hour (SD = 4 min/h). A total of 809 guardians completed the guardian’s questionnaire. Of them, 88% were mothers and 57% of respondents completed the questionnaire online (*N* = 461). Table 1 presents the mean and standard deviation for continuous explanatory variables and percentages for categorical explanatory variables.

Table 2 presents the results of the first models. All the 29 potential explanatory factors were included individually in the analyses. These first models indicated that being a boy (*p* = 0.000) and having high surgency temperament characteristics (*p* = 0.000) were associated with lower ST, whereas having high levels of effortful control was associated with higher ST(*p* = 0.000). Regarding the home setting, the following factors were associated with children’s lower ST: parental self-efficacy for activating child for PA (*p* = 0.036) and frequent visits together in PA places (*p* = 0.002). Parental perceived barriers related to children’s outside PA (*p* = 0.001) was associated with children’s higher ST.

**Table 2:** Associations of each explanatory factor and children’s sedentary time (min/h) in multilevel linear regression models

Level of influence	Variable name		β	Lower 95% CI	Upper 95% CI	*p*-value	N
Individual level	Gender		−0.98	−1.47	− 0.49	0.000	718
	Age		0.09	−0.17	0.36	0.497	718
	BMI		−0.00	− 0.05	0.05	0.945	684
	Surgency		−0.81	−1.11	−0.50	0.000	658
	Negative affectivity		0.11	−0.20	0.41	0.497	658
	Effortful control		0.69	0.31	1.08	0.000	658
Home level							
Parental beliefs and attitudes	Parental self-efficacy for activating child for physical activity (PA)		−0.09	− 0.18	−0.01	0.036	709
	Parental self-efficacy for limiting children’s screen time		0.07	−0.00	0.13	0.052	712
	Parental attitude on importance of physical activity (ref: somewhat disagree)						
		Neither agree or disagree	−0.51	−2.53	1.51	0.619	715
		Somewhat agree	−0.63	−2.49	1.22	0.503	
		Strongly agree	−1.09	−2.95	0.76	0.248	
	Parental attitude on societal pressures regarding screen time		−0.00	−0.09	0.08	0.960	710
	Parental perceived barriers related to children’s outside PA		0.13	0.06	0.02	0.001	711
	Parental beliefs of unhealthy energy balance-related behaviors (EBRBs) as a problem		−0.07	−0.47	0.32	0.724	712
Parenting practices	Rules for limiting children’s screen time		0.43	−0.19	1.04	0.173	685
	Parental practice on allowing child to run around inside		0.09	−0.23	0.40	0.586	715
	Parental screen use in front of children (ref: high parental screen use)						
		Low	−0.47	−1.14	0.20	0.171	714
		Middle	−0.56	− 1.22	0.11	0.103	
	Parental physical activity in front of children		−0.09	−0.31	0.13	0.420	717
	Frequent co-visits in PA places		−0.12	−0.12	− 0.04	0.002	717
	Mother’s educational level (ref: highly educated)						
		Low	0.16	−0.53	0.84	0.499	711
		Middle	−0.21	−0.81	0.40	0.648	
Preschool level							
Principals’ beliefs and attitudes	Principals’ personal interest in health (ref: neither disagree or agree)						
		Somewhat agree	0.54	−0.22	1.29	0.162	654
		Strongly agree	1.07	0.44	1.70	0.001	
	Principals’ attitude on the importance of increasing children’s physical activity		0.02	−1.34	1.37	0.982	665
	Principals’ attitude on the importance of decreasing children’s sedentary time		−0.23	−2.24	1.77	0.820	665
	Principals’ self-efficacy for influencing children’s behaviors		−0.18	−0.81	0.45	0.570	638
	Principals’ beliefs of unhealthy EBRBs as a problem		0.81	0.12	1.51	0.021	659
Organizational policies and practices	Frequency of visits in PA places		−0.19	−0.37	− 0.01	0.047	665
	Screen-time policy		0.16	−0.03	0.35	0.103	665
	Guidance for families policy		−0.21	− 0.38	−0.04	0.039	665
	Healthy PA policy		0.06	−0.04	0.13	0.200	665
	Active play possibility during free play		0.29	−0.48	1.06	0.466	674
Preschool neighborhood socioeconomic status (SES) (ref: high neighborhood SES)	Low		0.62	−0.65	1.89	0.339	718
	Middle		0.32	−0.73	1.37	0.554	

*CI* Confidence interval, *PA* Physical activity, *EBRBs* Energy balance-related behaviors, *SES* Socioeconomic status

Regarding the preschool setting, the following factors were associated with children’s lower ST: more frequent visits in PA places (*p* = 0.047) and written policies about guidance for families (*p* = 0.039). Preschool principals’ personal interest in health (*p* = 0.001 in the comparison between strongly agree and neither agree or disagree) and principals’ beliefs of unhealthy EBRBs as a problem (*p* = 0.021) were associated with children’s higher ST.

Table 3 presents the results of the final models. Analysis of these models included the explanatory factors that showed at least some evidence of an association with the previous phase (total 11 factors). Of the individual level factors, being a boy (*p* < 0.001) and having high levels of surgency-type temperament (*p* < 0.001) was associated with children’s lower ST. Regarding the home setting, more frequent visits together in PA places (*p* = 0.014) was associated with children’s lower ST, whereas parental attitude about barriers related to children’s physical activity outside (*p* = 0.032) was associated with children’s higher ST. None of the preschool-level factors remained significant.

**Table 3:** Final models: associations of explanatory factors and children’s sedentary time (min/h) in multilevel linear regression models

Level of influence	Variable name		β	Lower 95% CI	Upper 95% CI	*p*-value
Individual level	Gender		−1.07	−1.61	−0.52	0.001
	Surgency		−0.60	−0.92	− 0.27	0.001
	Effortful control		0.24	−0.16	0.64	0.232
Home level						
Parental beliefs and attitudes	Parental self-efficacy for activating children to physical activity (PA)		0.18	−0.24	0.61	0.403
	Parental self-efficacy for limiting children’s screen time		0.35	−0.05	0.74	0.085
	Parental perceived barriers related to children’s outside PA		0.35	0.03	0.67	0.032
Parenting practices	Frequent co-visits in PA places		−0.14	−0.26	−0.03	0.014
Preschool level						
Principals’ beliefs and attitudes	Principals’ personal interest in health (ref: neither agree or disagree)					
		Somewhat agree	−0.18	−3.58	3.21	0.916
		Strongly agree	0.26	−3.11	3.64	0.879
	Principals’ beliefs of unhealthy energy balance-related behaviors (EBRBs) as a problem		0.70	−0.36	1.76	0.200
Organizational policies and practices	Frequency of visits in PA places		−0.20	−0.44	0.04	0.104
	Guidance for families policy		0.05	−0.35	0.45	0.817
Additional fixed factors	Preschool time		−0.02	−0.05	0.02	0.331
	Measurement season		2.71	1.77	3.65	0.001

*CI* Confidence interval, *PA* Physical activity, *EBRBs* Energy balance-related behaviors, *SES* Socioeconomic status

Fixed effects in the final model explained 29.8% of the variance (marginal R^2^) in children’s ST. The proportion of variance explained in the model, including all fixed effects plus the random effect, was 53.5%, which indicates that the random factor (preschool) caused much of the total variance. Table 4 shows the 29.8% of marginal variance separately for individual-, home- and preschool-levels. Approximately 11% of the variance in children’s ST was attributed to factors related to the individual level; approximately 5% was attributed to home-level factors; and approximately 2% was attributed to preschool-level factors. Of the additional fixed factors, approximately 12% was attributed to the study season, and about 0.3% was attributed to the children’s average attendance at preschool.

**Table 4:** Percentage of variance explained by variance between fixed factors or by variance in full model

Level of influence	Variable name	% explained variance in full model
Individual level factors	Gender	10.7%
	Surgency	
	Effortful control	
Home level factors	Parental self-efficacy for activating child to physical activity (PA)	5.3%
	Parental self-efficacy for limiting children’s screen time	
	Parental perceived barriers related to children’s outside PA	
	Frequent co-visits in PA places	
Preschool level factors	Principals’ personal interest in health	1.7%
	Principals’ beliefs of unhealthy energy balance-related behaviors (EBRBs) as a problem	
	Frequency of visits in PA places	
	Guidance for families policy	
Additional fixed factors	Preschool time	0.3%
	Measurement season	11.7%

## Discussion

The aim of this study was to examine individual-, home- and preschool level- factors associated with preschool children’s ST. Additionally, it was studied how much individual-, home- and preschool- level factors explained the variance in children’s ST. Of the individual level factors, being a boy and having a surgency-type temperament was associated with lower ST**.** Of the home-level factors, parental attitude about barriers related to children’s PA outside was associated with children’s higher ST, whereas more frequent visits together in PA places was associated with children’s lower ST. None of the measured preschool-level factors remained significant in the final models. Together, these results suggest that individual-level factors explain more the variance in children’s ST than home and preschool settings. Notably, the proportion of variance explained in ST, which included all fixed effects plus the random effect, was approximately 54%. This high percentage indicates that the random factor (preschool) captured substantial amounts of additional variance. However, the variance of preschool factors used in this study remained low. This difference indicates that we may not have measured the relevant preschool factors in our study to fully explain the potential variance in children’s ST.

Two individual-level factors, namely being a boy and having high levels of surgency, were associated with lower ST. The individual-level factors also explained most of the variance in children’s ST. Our findings support the results of many other studies: boys have less ST than girls [1]. Two recent studies concluded that activity temperament was associated with lower ST [52] whereas anxious temperament was associated with higher ST [53]. The studies also indicated that mothers with low income or who are overweight particularly dealt with children’s challenging temperament traits by allowing their children use the television [54, 55]. In addition, Gubbels et al [14, 53] recognized that gender or temperament moderated the association between a preschool setting and children’s movement behaviors. These previous studies have used different temperament instrument than this study. However, similar to our findings, these results underline that children’s temperament influences their willingness to engage in movement behaviors already very early in life. Consequently, an improved strategy may involve better recognizing children’s individual needs and developing intervention strategies that are tailored to their specific characteristics.

To our knowledge, no previous studies have included factors in both preschool and home setting in the same study as we did, and therefore making comparisons to previous studies is difficult. However, Schmutz et al stated that environmental factors, for example, children living in a dual-guardian household, concerns of neighborhood safety, apartment size and guardians with no sports-club membership explained approximately 17% of the variance in children’s ST [52]. On the contrary, Hnatiuk et al found no other home-level factor associated with three-to-four-year-old children’s ST, except a father’s television viewing before 6 pm [56]. However, no preschool-level factors were included in these studies. Nonetheless, Hesketh et al stated that British preschool explained little variance in children’s anthropometry [57], and other studies had stated that a preschool setting explained 10 to 50% variance in children’s PA [58–61]. However, less is known about variance in children’s ST explained by preschool setting. Preschool care in Finland is widely regulated and standardized, ensuring similar levels of care for all children. Therefore, the preschool setting possibly has little influence in explaining the variance in children’s ST. In addition, each setting may differ if ST is a personal choice or ST has environmental and social constraints [62]. Preschool setting may have multiple social norms for sitting and expectations for children to sit, for instance, morning circles, regular meal times or puzzle sessions [42], which are quite similar across preschools. Thus, few opportunities may exist for children’s individual choice, causing little variance in children’s ST.

Most of the factors in preschool settings were measured from the perspective of principals, but how well these items capture the daily practices conducted in preschool is questionable. For instance, various policies were measured from the perspective of principals; however, how aware early educators are of these policies is unknown, along with how much these policies are actually implemented in daily practices. It may therefore be that the questionnaire for principals captured inadequately the variables that were aimed to measure due to the difficulty in operationalizing the terms of attitudes, beliefs and policies. Overall, our results support the need to study other potential factors associated with children’s ST in the preschool setting. In addition, of all the factors included in this study, study season had the highest percentage of variance. Evidence indicates seasonal variation in children’s ST and PA, that is, children are more sedentary in rainy and dark weather during autumn and winter time [63–65]. This data was collected between early autumn and early spring, covering a wide variety of weather conditions.

Another research finding related to this theme was the association between parental perceived barriers related to children’s outside PA and children’s higher ST. The items included in this sum variable related to poor weather or difficulty of supervising a child outside. Previous qualitative studies have also highlighted parental attitudes on how poor weather, namely cold or rainy weather, limits children’s PA and how a child is not allowed to play outside alone for safety reasons [66]. We did not measure in detail what type of weather parents consider is poor weather; however, the development of intervention strategies to account for these parental concerns regarding the weather or outside supervision is important as these concerns may limit children’s possibilities to be active.

One study finding concerned the association between having frequent practices of visiting PA places with children in the home setting and lower children’s ST. Similarly, frequent visits in PA places in the preschool setting was associated with lower children’s ST in the individual models, although this association did not remain statistically significant in the final models. Overall, this practice of frequent visits to PA places may be a good target when developing intervention strategies aiming to reduce children’s ST. Being active in PA places may provide better possibilities for children to have unstructured free play that allows them to move around, and therefore is associated with lower ST. Regular co-participation in PA, either inside or outside, benefits the development of children’s social, self-regulation and motor skills, but also naturally provides healthy role-modelling opportunities to caregivers [67]. Most of the items included in these visiting PA places variables were activities conducted outside, for instance, in yards, playgrounds and nature. Previous studies have identified that time outdoors is associated with lower children’s ST [68, 69]. This study did not find any significant associations between SES factors and children’s ST, which supports the findings of other studies that measured ST by objective measurement [24]. However, mediators (intervening factors) may exist between the associations of SES and children’s ST. Conducting mediational analyses, despite non-significant overall associations, may enable an easier understanding of the mechanisms of influence [70]. In addition, some SES differences may exist in the presented co-participation practices. For instance, the previous study using this same study sample [71] found out that guardians with a low educational background reported more frequent visits with their child to their own yard, whereas guardians with high educational backgrounds reported more frequent visits with their children to indoor sports facilities. However, only frequent co-participation in own yard acted as mediator in the associations between guardians with a low educational background and children’s ST. [71]

The current study has several strengths and limitations that should be accounted for. Multiple items that were similarly asked from the perspective of both guardians and preschools were measured. However, some of the measured items were not validated, and had quite low Cronbach alpha. In addition, other important factors that influence children’s ST may exist, such as early educators’ motivation and self-efficacy. For instance, previous study using this same study sample found out that early educators’ practice often interrupting children’s ST, more frequent physical activity (PA) theme weeks, and higher number of physical education (PE) lessons were associated with children’s lower ST in preschools [72]. Our study did not cover physical environment factors either in home or preschool settings, which have most often been associated with children’s ST at least in preschool setting [20]. However, multiple factors that are rarely measured were included. Compared to other studies among preschool-aged children, the sample size of this study was quite high and covered a variety of preschools around Finland. While a hip-placed accelerometer can provide ST over a prolonged period, they are less valid in distinguishing sedentary postures, such as lying and sitting, from other light-intensity activities performed while standing. Different definitions of ST, different used cut-points and different data reduction methods may explain some of the dissimilarity between our results and previous studies. In addition, it may be relevant to measure children’s ST with multiple methods. Proxy-reports and observations may provide valuable additional knowledge of the specific SBs (e.g. reading, screen time) that cannot be captured with accelerometers. This knowledge is important as the correlates of specific SBs seem to be different than that of overall ST [22]. Our data is cross-sectional, which prevents drawing causal inferences. The detected associations between explanatory factors and outcome were relatively small. It may be that it was failed to find associations when one existed due to a lack of power and sample size. However, by including multiple levels of influence, especially using both home and preschool settings within the same study, our study introduced novel information about factors explaining the variance in preschool children’s ST.

## Conclusion

This study recognized multiple factors on individual, home and preschool levels that were associated with preschool children’s ST. Children’s individual characteristics such as gender and temperament were associated with children’s ST, and explained more the variance in children’s ST than settings.. Future strategies aiming to reduce children’s ST should consequently tailor intervention strategies to children’s individual characteristics, without forgetting the influence of home and preschool settings. The preschool-level factors included in this study explained little the variance in children’s ST between preschools, possibly because the included measures did not capture the essential factors in preschool setting that influence children’s ST. In conclusion,the development of improved methods of measuring the preschool-level factors is essential.

## Data Availability

Researchers interested in the data from this study may contact the principal investigator Eva Roos at eva.roos@folkhalsan.fi.

## Abbreviations

DAGIS: Increased health and wellbeing in preschools
EBRBs: Energy balance-related behaviors
PA: Physical activity
SB: Sedentary behavior
SD: Standard deviation
SES: Socioeconomic status
ST: Sedentary time
